# Corrigendum: Predicting T Cell Receptor Antigen Specificity From Structural Features Derived From Homology Models of Receptor-Peptide-Major Histocompatibility Complexes

**DOI:** 10.3389/fphys.2021.790998

**Published:** 2022-01-24

**Authors:** Martina Milighetti, John Shawe-Taylor, Benny Chain

**Affiliations:** ^1^Division of Infection and Immunity, University College London, London, United Kingdom; ^2^Cancer Institute, University College London, London, United Kingdom; ^3^Department of Computer Science, University College London, London, United Kingdom

**Keywords:** T cell receptor, antigen prediction, TCR-pMHC binding, homology modelling, T cell

In the original article, there was a mistake in the provided supplementary material for the datasets Dash (Dash et al., 2017), 10X (10XGenomics, 2020), and newVdj (Bagaev et al., 2020) as published. The mistake derived from an imprecision in the method used to reconstruct the complete amino sequence for the TCRs from the V/J gene and CDR3 information, which impacted a small proportion of the sequences in these sets (between 0.5 and 7% depending on the set). The wrong sequences have now been either removed or corrected. The corrected version of these datasets is provided as supplementary material.

The authors apologize for this error and state that this does not change the scientific conclusions of the article in any way. The original article has been updated.

## Publisher's Note

All claims expressed in this article are solely those of the authors and do not necessarily represent those of their affiliated organizations, or those of the publisher, the editors and the reviewers. Any product that may be evaluated in this article, or claim that may be made by its manufacturer, is not guaranteed or endorsed by the publisher.
