# Supplementary material for: Predicting T Cell Receptor Antigen Specificity From Structural Features Derived From Homology Models of Receptor-Peptide-Major Histocompatibility Complexes
Source: Front Physiol. 2021 Sep 8;12:730908. doi: 10.3389/fphys.2021.730908 (PMC8456106; doi:10.3389/fphys.2021.730908)
Supplement: Supplementary file 2 [file Data_Sheet_2.pdf]

# Supplementary Material

## 1 SUPPLEMENTARY DATA

The following are supplied as supplementary materials:

1. Sequences for all the datasets used, specifically:
   - **sequences from STCRDab PDB files** - these are the sequences from the PDB files used for the initial feature extraction
   - **STCRDab set metadata** - metadata associated with the sequences from the STCRDab
   - **10XGenomics set sequences** - sequences for the structures included in the 10X set
   - **experimental constructs sequences** - sequences for the structures included in the expt set
   - **Dash set** - sequences for the structures included in the Dash set
   - **ATLAS sequences** - sequences for the structures included in the TCR ATLAS set, including the affinity information from the ATLAS
   - **VDJDb validation sequences** - sequences for the structures included in the new VDJDb set
2. Results file for 10-fold CV, both for Figure 6 and for Figure 8b.
3. All result files with decision function scores for each TCR-peptide pair. A README file is included with filename explanations.

## 2 SUPPLEMENTARY TABLES AND FIGURES

**Table S1.** Number of complexes included in the classification for each of the cases.

| | **40** | **60** | **80** | **100** |
|---|---|---|---|---|
| **TCR** | 390 | 401 | 401 | 404 |
| **pMHC** | 404 | 404 | 404 | 404 |
| **Complex** | 280 | 404 | 404 | 404 |
| **All** | 269 | 401 | 401 | 401 |

**Table S2. Results of benchmarking on single epitopes.** For each epitope, the performance of each tool is calculated (ROC AUC). In each row, the best-performing tool is highlighted in bold.

| | N pos | N neg | in_pdb | in_vdjdb | distances | dist-atr | atchley | atchley-dist | atchley-dist-atr | ImRex | ERGO LSTM | ERGO AE |
|---|---|---|---|---|---|---|---|---|---|---|---|---|
| **ALYGFVPVL** | 5 | 121 | no | no | 0.618 | 0.516 | 0.460 | 0.499 | 0.488 | 0.471 | 0.293 | **0.660** |
| **APARLERRHSA** | 3 | 124 | no | no | 0.508 | 0.532 | 0.503 | 0.505 | 0.516 | **0.901** | 0.591 | 0.562 |
| **HMTEVVRHC** | 4 | 121 | no | no | 0.455 | 0.597 | **0.800** | 0.545 | 0.622 | 0.678 | 0.543 | 0.502 |
| **NLNCCSVPV** | 4 | 123 | no | no | **0.717** | 0.650 | 0.396 | 0.681 | 0.640 | 0.547 | 0.677 | 0.567 |
| **RLARLALVL** | 5 | 122 | no | no | 0.233 | 0.303 | 0.457 | 0.179 | 0.249 | 0.433 | 0.575 | **0.582** |
| **SSCMGGMNWR** | 3 | 124 | no | no | 0.745 | **0.952** | 0.806 | 0.790 | 0.914 | 0.618 | 0.487 | 0.309 |
| **VVMSWAPPV** | 7 | 120 | no | no | 0.395 | 0.538 | 0.548 | 0.362 | **0.555** | 0.482 | 0.461 | 0.433 |
| **AVFDRKSDAK** | 175 | 869 | no | yes | 0.469 | 0.444 | 0.501 | 0.461 | 0.440 | 0.534 | **0.716** | 0.669 |
| **AYAQKIFKI** | 4 | 62 | no | yes | 0.750 | 0.395 | 0.532 | 0.746 | 0.403 | 0.266 | 0.690 | **0.867** |
| **FLASKIGRLV** | 3 | 24 | no | yes | 0.444 | 0.667 | 0.431 | 0.333 | 0.653 | 0.542 | **1.000** | 0.597 |
| **FLYALALLL** | 7 | 9 | no | yes | 0.540 | 0.683 | 0.111 | 0.476 | 0.254 | 0.349 | **1.000** | 0.968 |
| **HGIRNASFI** | 139 | 1673 | no | yes | 0.523 | 0.669 | 0.477 | 0.502 | 0.626 | 0.607 | **0.926** | 0.917 |
| **IVTDFSVIK** | 207 | 421 | no | yes | 0.549 | 0.613 | 0.655 | 0.630 | 0.646 | 0.668 | **0.821** | 0.795 |
| **KLGGALQAK** | 324 | 2161 | no | yes | 0.488 | 0.480 | 0.526 | 0.494 | 0.495 | 0.511 | **0.739** | 0.630 |
| **KTWGQYWQV** | 3 | 10 | no | yes | 0.900 | 0.633 | 0.600 | 0.867 | 0.567 | 0.433 | **1.000** | 0.933 |
| **LLDFVRFMGV** | 10 | 18 | no | yes | 0.789 | 0.639 | 0.372 | 0.639 | 0.517 | 0.294 | 0.767 | **0.800** |
| **LSLRNPILV** | 64 | 1796 | no | yes | 0.419 | 0.444 | 0.586 | 0.489 | 0.454 | 0.520 | **0.902** | 0.745 |
| **MLDLQPETT** | 6 | 6 | no | yes | 0.556 | 0.389 | 0.333 | 0.583 | 0.222 | **0.778** | 0.694 | 0.583 |
| **RAKFKQLL** | 77 | 169 | no | yes | 0.640 | 0.534 | 0.586 | 0.639 | 0.532 | 0.554 | 0.725 | **0.726** |
| **RLRAEAQVK** | 57 | 336 | no | yes | 0.465 | 0.412 | 0.541 | 0.465 | 0.433 | 0.538 | **0.753** | 0.727 |
| **RMFPNAPYL** | 4 | 12 | no | yes | 0.458 | 0.563 | 0.646 | 0.458 | 0.667 | 0.542 | **0.958** | 0.750 |
| **SLFNTVATLY** | 5 | 34 | no | yes | 0.312 | 0.394 | 0.176 | 0.312 | 0.512 | 0.435 | **0.771** | 0.712 |
| **SSPPMFRV** | 20 | 1795 | no | yes | 0.481 | 0.410 | 0.465 | 0.387 | 0.422 | 0.665 | **0.891** | 0.814 |
| **SSYRRPVGI** | 455 | 1390 | no | yes | 0.425 | 0.470 | 0.543 | 0.508 | 0.467 | 0.282 | **0.938** | 0.927 |
| **TVYGFCLL** | 46 | 1839 | no | yes | 0.395 | 0.435 | 0.364 | 0.346 | 0.403 | 0.453 | **0.915** | 0.757 |
| **ASNENMETM** | 161 | 1717 | yes | yes | 0.505 | 0.597 | 0.516 | 0.441 | 0.604 | 0.486 | **0.948** | 0.900 |
| **ELAGIGILTV** | 178 | 347 | yes | yes | 0.740 | 0.734 | 0.831 | 0.776 | 0.755 | 0.575 | **0.862** | 0.756 |
| **FLRGRAYGL** | 32 | 11 | yes | yes | 0.139 | 0.131 | **0.787** | 0.347 | 0.122 | 0.665 | 0.585 | 0.531 |
| **GILGFVFTL** | 532 | 2031 | yes | yes | 0.718 | 0.738 | 0.831 | 0.777 | 0.782 | 0.823 | **0.982** | 0.969 |
| **GLCTLVAML** | 98 | 1850 | yes | yes | 0.728 | 0.727 | 0.763 | 0.750 | 0.744 | 0.756 | **0.991** | 0.980 |
| **LGYGFVNYI** | 4 | 10 | yes | yes | 0.925 | 0.850 | **1.000** | **1.000** | 0.925 | 0.925 | **1.000** | 0.925 |
| **LLFGYPVYV** | 85 | 35 | yes | yes | 0.874 | 0.894 | 0.890 | **0.901** | 0.887 | 0.873 | 0.882 | 0.881 |
| **NLVPMVATV** | 63 | 1878 | yes | yes | 0.661 | 0.659 | 0.550 | 0.638 | 0.623 | 0.495 | **0.987** | 0.956 |
| **SSLENFRAYV** | 147 | 1615 | yes | yes | 0.536 | 0.580 | 0.536 | 0.508 | 0.541 | 0.630 | **0.836** | 0.730 |

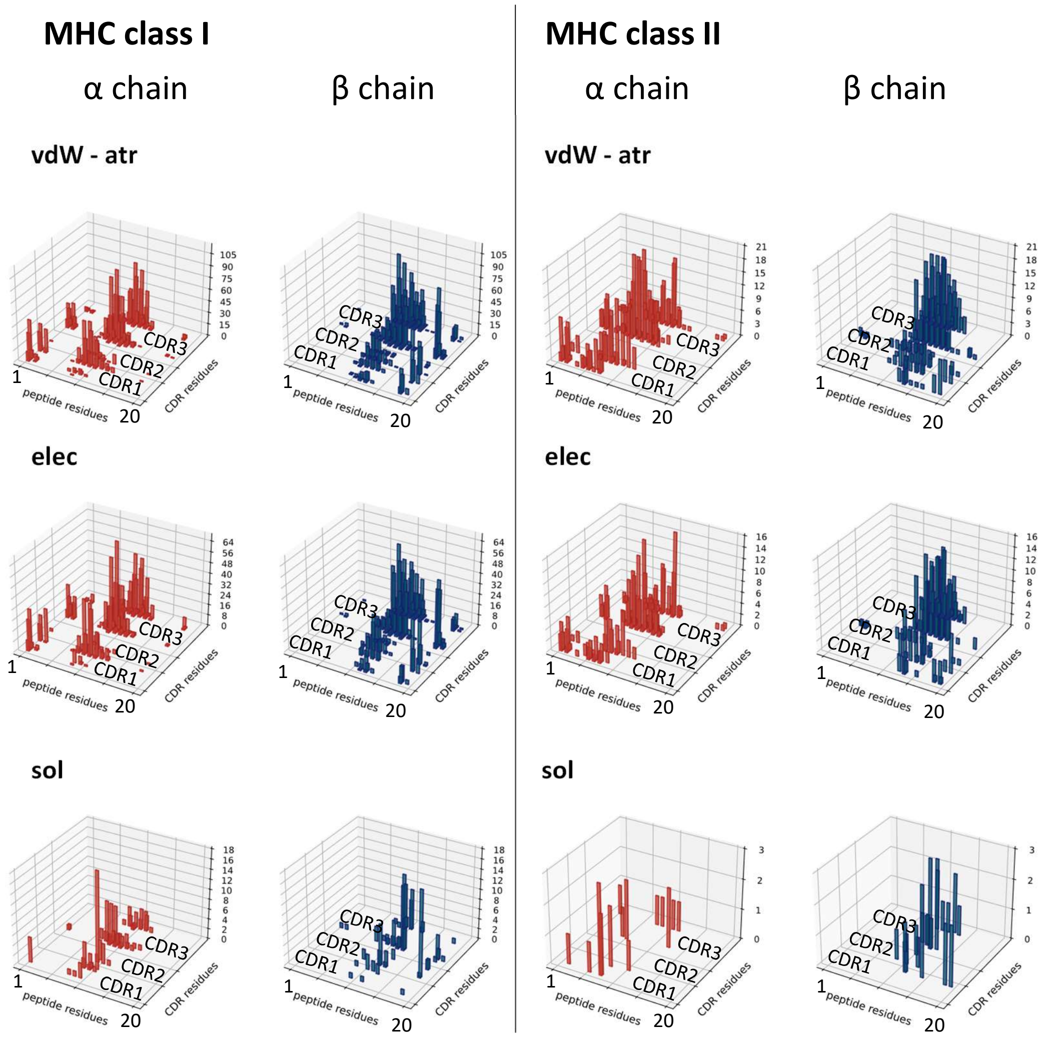


**Figure S1. Energy interactions for class I and class II complexes** Analogous to Figure 1c, but for all energy feature sets. The histograms show the number of structures that make a favourable contact (energy $< 0$). Repulsive vdW excluded as this component is always $> 0$.

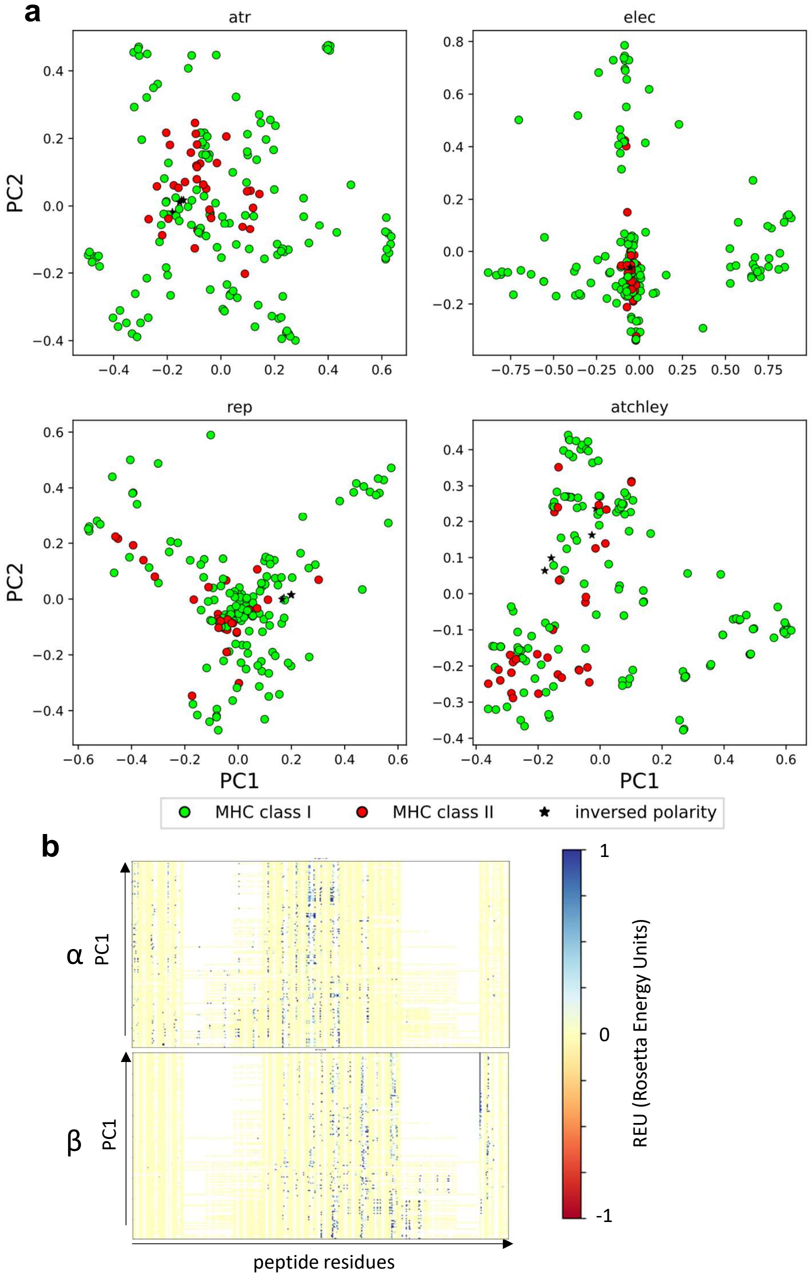


**Figure S2. PCA on all extracted features. (A)** PCA for feature sets not included in Figure 2a. Class I and class II complexes are shown in green and red, respectively. The stars indicate the structures that have been reported to have inversed polarity (i.e. the TCRs bind the pMHC complex at 180 degree angle). **(B)** Linearised vectors used for the solvent energy PCA, ordered according to their PC1 score. On the x-axis, the calculated solvent energy between each CDR residue and each peptide residue (27-1, 28-1,...,116-1, 117-1, 27-2,...,117-20). Analogous to Figure 2b.

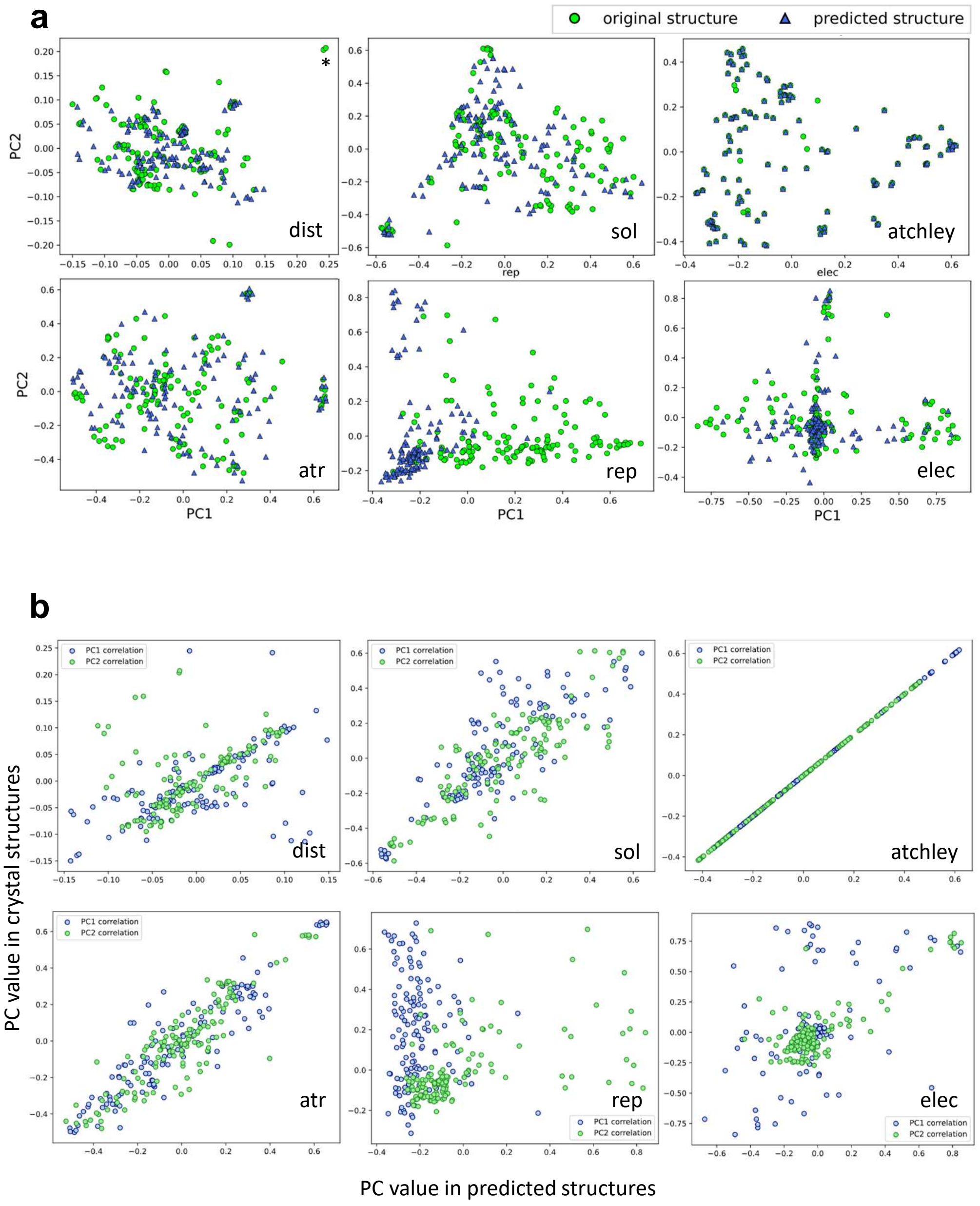


**Figure S3. PCA of original vs predicted. (A)** PCA for each set showing overlay between original and predicted structures. Asterisks (*) in the distance plot indicates the inversed polarity structures. **(B)** Correlation for PC1 and PC2 values between original and predicted structures. Each blue dot is a complex and has (x,y) coordinates that depend on PC1 values for predicted and original structure. Similarly for PC2 (green dots).

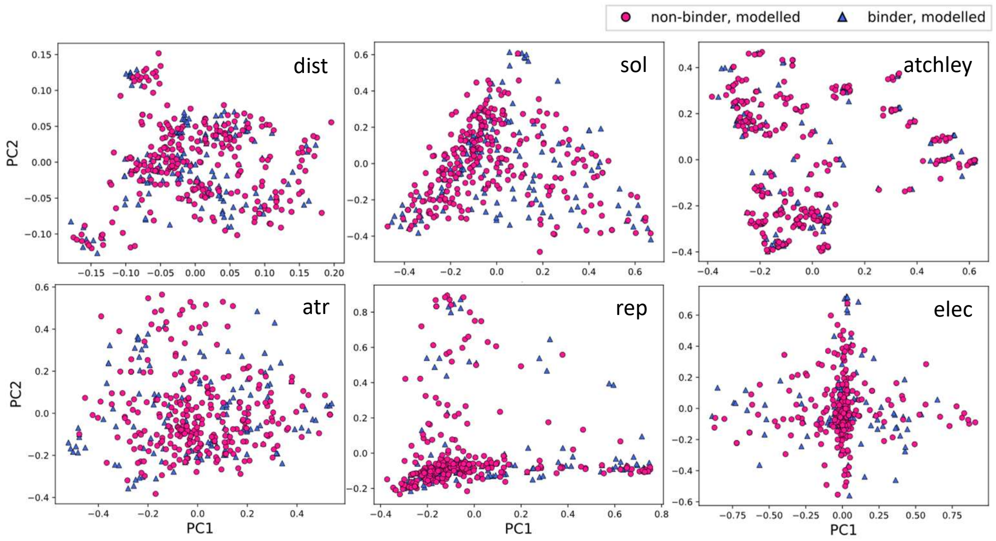


**Figure S4. PCA of original vs predicted and of binding vs non-binding.** PCA for each set showing overlay of binding and non-binding complexes (predicted structures, blue triangles and magenta circles, respectively).

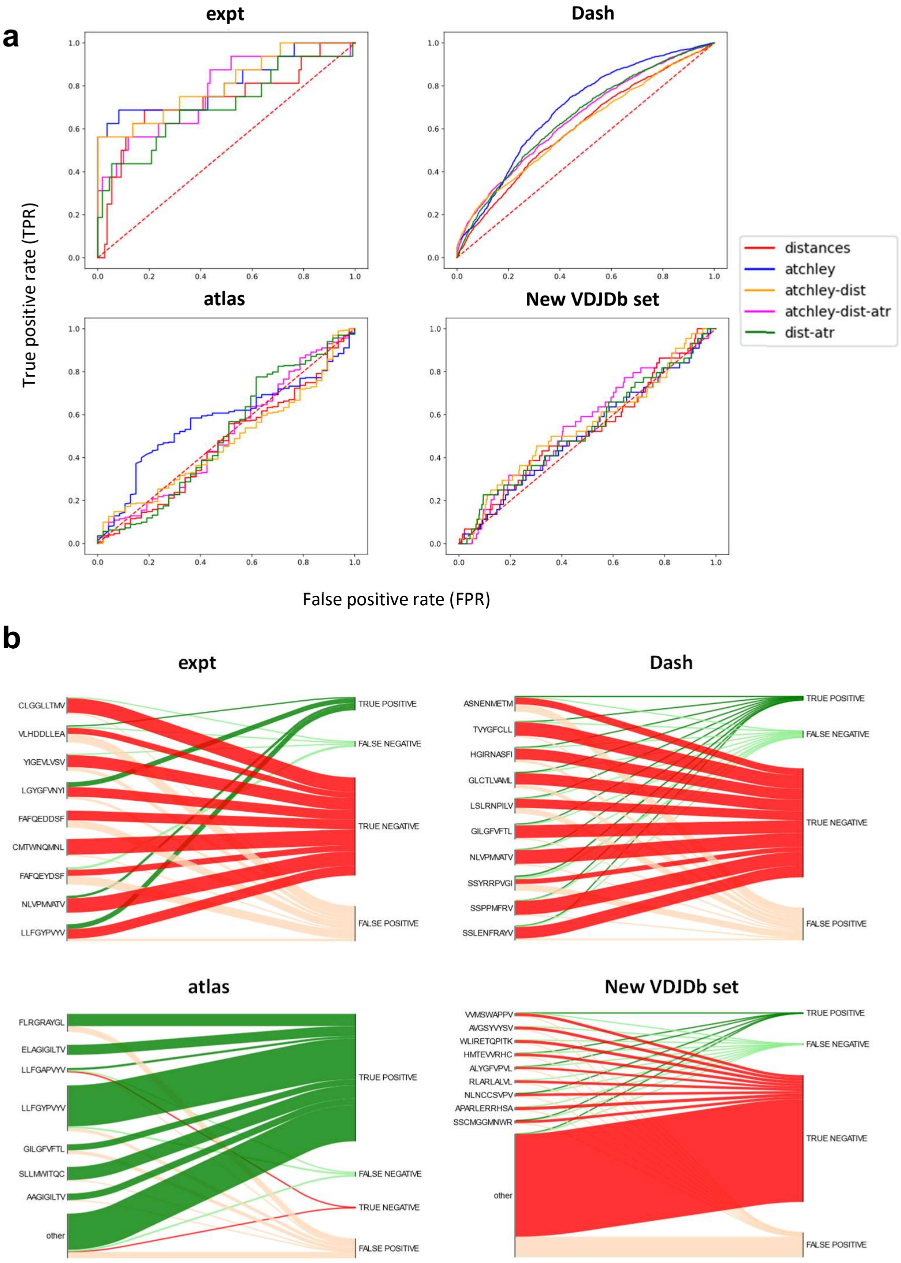


**Figure S5. Results of all validation sets used. (A)** ROC curves obtained when the model trained on the STCRDab set are used for prediction on each of the validation sets. **(B)** For the model trained on STCRDab using distances only, the diagram shows which proportion of examples from each epitope are classified correctly (true positives and true negatives) or incorrectly (false positives and false negatives) for each of the validation sets used. This is shown in the form of a Sankey diagram, where from each epitope annotated on the left-hand side of the plot, a line is drawn for each TCR recognising that specific epitope to the final result for the complex (correctly classified as binder or non-binder, or incorrectly classified). The width of each section is proportional to the number of complexes that follow that classification.

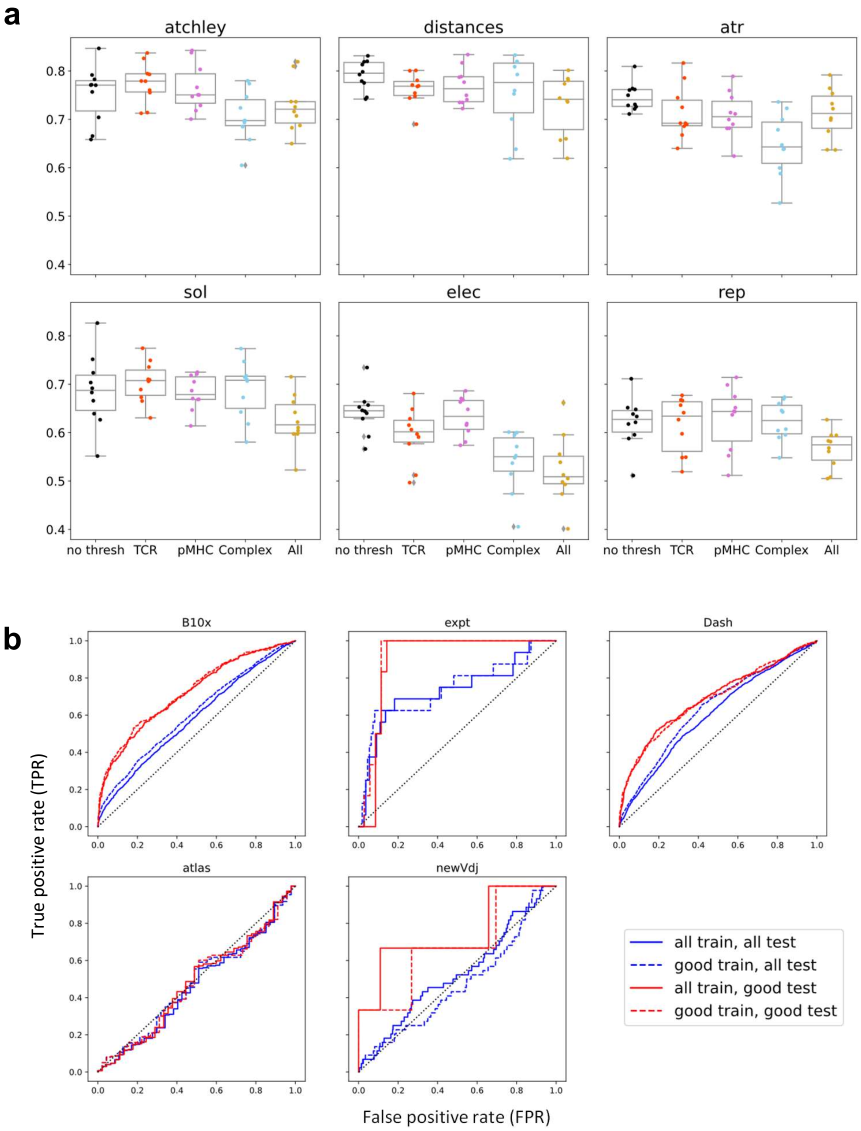


**Figure S6. Effect of homology modelling template selection. (A)** For each feature set, results on 10-fold CV are shown. The structures were modelled by setting no threshold on the homology modelling template selection (the no thresh category is the same as in Figure 6a and c). For the TCR, pMHC and Complex category, a threshold of 40% was set to the homology modelling template selection (meaning that no more than 40% sequence homology was allowed with the best template under the multi-weighted scheme, as described in Jensen et al. (2019)). Finally, in the All category, a threshold was set for selection of template of TCR, pMHC and Complex simultaneously. **(B)** Performance of each of the validation set when the model is trained on the entire STCRDab set (all train) or only the STCRDab structures with good templates (as defined in methods - good train), and when predictions are made on all complexes (all test) or only complexes with good templates (good test).

## REFERENCES

Jensen KK, Rantos V, Jappe C, Olsen H, Closter Jespersen M, Jurtz V, et al. TCRpMHCmodels: Structural modelling of tcR-pMHc class i complexes. *Scientific Reports* **9** (2019). doi:10.1038/s41598-019-50932-4.
